# Tractography Delineates Microstructural Changes in the Trigeminal Nerve after Focal Radiosurgery for Trigeminal Neuralgia

**DOI:** 10.1371/journal.pone.0032745

**Published:** 2012-03-06

**Authors:** Mojgan Hodaie, David Qixiang Chen, Jessica Quan, Normand Laperriere

**Affiliations:** 1 Division of Neurosurgery, Toronto Western Hospital and University of Toronto, Toronto, Ontario, Canada; 2 Faculty of Medicine, Institute of Medical Science, University of Toronto, Toronto, Ontario, Canada; 3 Division of Neurosurgery, University of Toronto, Toronto, Ontario, Canada; 4 Radiation Medicine Program, Princess Margaret Hospital and University of Toronto, Toronto, Ontario, Canada; University of Maryland, College Park, United States of America

## Abstract

**Purpose:**

Focal radiosurgery is a common treatment modality for trigeminal neuralgia (TN), a neuropathic facial pain condition. Assessment of treatment effectiveness is primarily clinical, given the paucity of investigational tools to assess trigeminal nerve changes. Since diffusion tensor imaging (DTI) provides information on white matter microstructure, we explored the feasibility of trigeminal nerve tractography and assessment of DTI parameters to study microstructural changes after treatment. We hypothesized that trigeminal tractography provides more information than 2D-MR imaging, allowing detection of unique, focal changes in the target area after radiosurgery. Changes in specific diffusivities may provide insight into the mechanism of action of radiosurgery on the trigeminal nerve.

**Methods and Materials:**

Five TN patients (4 females, 1 male, average age 67 years) treated with Gamma Knife radiosurgery, 80 Gy/100% isodose line underwent 3Tesla MR trigeminal nerve tractography before and sequentially up to fourteen months after treatment. Fractional anisotropy (FA), radial (RD) and axial (AD) diffusivities were calculated for the radiosurgical target area defined as the region-of-interest. Areas outside target and the contralateral nerve served as controls.

**Results:**

Trigeminal tractography accurately detected the radiosurgical target. Radiosurgery resulted in 47% drop in FA values at the target with no significant change in FA outside the target, demonstrating highly focal changes after treatment. RD but not AD changed markedly, suggesting that radiosurgery primarily affects myelin. Tractography was more sensitive than conventional gadolinium-enhanced post-treatment MR, since FA changes were detected regardless of trigeminal nerve enhancement. In subjects with long term follow-up, recovery of FA/RD correlated with pain recurrence.

**Conclusions:**

DTI parameters accurately detect the effects of focal radiosurgery on the trigeminal nerve, serving as an in vivo imaging tool to study TN. This study is a proof of principle for further assessment of DTI parameters to understand the pathophysiology of TN and treatment effects.

## Introduction

TN is a chronic neuropathic facial pain in one or more trigeminal nerve divisions [1]. The most prevalent theory is ephaptic signal transmission secondary to neurovascular compression at the nerve entry zone [2], [3], however focal areas of demyelination have also been implicated [3], [4]. Surgical treatment, including microvascular decompression (MVD), rhizotomy or focal radiosurgery, is undertaken if medication is insufficient or there are undesirable side-effects. The most common type of focal radiosurgery for trigeminal neuralgia consists in Gamma Knife radiosurgery (GKRS).

GKRS consists in delivery of highly focused ^60^Co radiation to the the trigeminal nerve, outside the root entry zone in the brainstem [5]. The exact position of the shot varies ranging from just outside the nerve entry zone [6] to the distal segment of the trigeminal nerve [7]. Despite widespread clinical use of GKRS for the treatment of TN, there are no objective imaging methods to adequately correlate treatment and outcome, particularly since TN GKRS is associated with delay in pain relief [8] and a relatively lower risk of facial dysesthesia compared with other TN treatments [9], although more recent reports demonstrate an increasing trend of facial dysesthesiae with time [10]–[12]. Lack of clinical benefit may correlate with either lack of effectiveness or targeting inaccuracy. Current imaging modalities rely primarily on comparison of pre and post MR imaging with the addition of gadolinium-enhancement. The latter shows enhancement on the trigeminal nerve in approximately 80% of cases, however this finding is neither predictive of benefit nor recurrence of pain [13], [14]. Clearly, better methods of assessment of changes in the trigeminal nerve after GKRS are needed.

Neither the effect of GKRS nor the mechanisms underlying the resultant analgesic effect are well understood. Axonal degeneration has been proposed as a possible mechanism based on animal studies [15], as has demyelination [4]. Whatever the effect, the mechanism may not necessarily relate to permanent nerve injury, since in a significant proportion of cases the trigeminal nerve function remains clinically unchanged. Further, recurrence of pain implies recovery from radiation injury and points away from permanent changes. The effect of GKRS is also dose-dependent. The typical dose of 80 Gy is subnecrotic [15]. Increasing dose beyond 80 Gy is associated with increasing risk of facial numbness and at 100 Gy frank necrosis can be observed [15]. The radiosurgical dose has a sharp drop-off implying a much lower dose immediately outside the target. This highly focal area of injury is characteristic of GKRS [16].

White matter tractography is gaining increasing application in imaging. In addition to showing a 3D representation of fiber tracts, water anisotropy is susceptible to changes in neural microstructure, and allows for the detection of such changes in an in vivo system. It is now accepted that DTI parameters are sensitive to microstructural changes since processes including demyelination and axonal injury can affect water anisotropy [17], [18]. Recent technical advances are overcoming the challenges of imaging small white matter bundles, such as the cranial nerves [19]. We employed this technique to study the hypothesis that a) trigeminal nerve tractography can accurately detect focal changes in the trigeminal nerve after GKRS and is more informative than the standard 2D MR image post-treatment and b) tractography can detail microstructural changes that occur in the nerve after focal radiosurgery, and provide further information on the possible mechanism of action of GKRS in TN. To test our hypothesis, trigeminal tracts were reconstructed in five subjects pre and post-radiosurgery and compared with standard post-treatment 2D MR imaging. In each instance, DTI parameters including fractional anisotropy (FA), radial (RD) and axial (AD) diffusivities were measured before and after GKRS and changes were analyzed.

## Methods

### Ethics Statement

This study involved retrospective analysis of MR data. Informed consent was not requested by our ethics board since the study did not involve any specific participation of subjects in the study. MR data was anonymized prior to analysis. Institutional review board (University Health Network Research Ethics Board) approval was obtained for this study. The University Health Network Research Ethics Board does not require informed consent when studies involve analysis of retrospective data, regardless of whether this is clinical or imaging (MRI) data analysis. All patient information is stored in secure databases, only accessed upon approval of a research study. Our hospital and research ethics boards (University Health Network Research Ethics Board) does not require that patients give written consent for their information to be stored in specific hospital databases. The merits of each research proposal are assessed separately by our institution's ethics board. Informed written consent is requested for prospective studies, or those that involve patient identifiers such that the study or its publication would interfere with patient confidentiality. This case does not apply to our current study.

Subjects consisted in five patients (4F, 1M) with classic TN, chosen on the basis of availability of pre and post treatment DTI studies and length of follow-up. Average age was 67 years. Three patients had left sided pain and two had right sided pain. All patients had prior treatment with medications, to which they were either intolerant, or the medications had poor effectiveness. The most common trigeminal distribution of pain was V2. Upon clinical assessment, none of the patients showed evidence of new trigeminal numbness after their radiosurgery treatment. Demographics and details for each patient are presented in Table 1. All subjects had TN that was not amenable to medical treatment alone. Subjects were treated with GKRS (Leksell Gamma Knife 4C unit ©, Stockholm, Sweden) using 80 Gy to the 100% isodose line, 4 mm collimator in a single fraction. The target was chosen as a point outside the nerve entry zone, such that the 20% isodose line remained outside the brainstem, and the 80% isodose line wrapped around the contour of the trigeminal nerve on coronal images. This method allowed us to constrain the dose to the brainstem, which was kept to less than 15 Gy to 1 mm^3^.

**Table 1:** Demographics and clinical presentation of subjects studied.

Subject/Gender	Age	Trigeminal division affected	Laterality	Medications	Effect of treatment	Post GKRS numbness	Post treatment MR gadolinium enhancement
**S1/F**	43	V1, V2, V3	Left	Intolerant/poor effectiveness	Significant improvement initially, pain recurred at 1 year	No	Yes
**S2/F**	77	V2, V3	Right	Carbamazepine 800 mg daily	No pain at one year post treatment	No	No
**S3/M**	53	V1, V2	Right	Carbamazepine 600 mg daily	No pain at 6 months	No	No
**S4/F**	82	V2	Left	Intolerant/poor effectiveness	No pain at 6 months	No	Yes
**S5/F**	83	V1	Left	Intolerant/poor effectiveness	No pain at 6 months	No	Yes

All patients underwent pre and serial post-treatment imaging at 6–7 months post treatment. Subjects S1, S2 had additional post-treatment imaging at 14 and 12 months respectively.

### Tractography Procedure

#### Data Acquisition, image processing and registration

3T MR imaging (GE Signa HDx) imaging sequences included T1 anatomical (axial Fast spoiled gradient echo (FSPGR). slice thickness = 1 mm, slice spacing = 1 mm, repetition time = 8.0 ms, echo time = 3.1 ms); diffusion weighted images (DWI) were acquired using an 8-channel head coil with DTI 25 directions Array special sensitivity encoding technique (ASSET) = 2, Echo Planar/Spin Echo sequence (3 mm thickness, 55 slices, 1 baseline, slice spacing = 0 mm, echo time = 86.6 ms, repetition time = 12,000 ms, NEX = 1). The acquisition matrix size was 128×128, and b-value = 1000 s/mm^2^. Images were processed, registered and tractography were performed using the 3D Slicer software (http://www.slicer.org, NA-MIC©) [20] following the procedure described elsewhere [19].

#### DTI Tractography

Tractography of bilateral trigeminal nerves was performed from both pre and post-treatment DTI Tensors. Streamline tractography was performed with initial seed spacing = 0.5, linear measure starting threshold = 0.2, fractional anisotropy threshold = 0.2, curvature threshold = 0.8 rad.

#### Diffusivity Data

Two distinct ROI on the trigeminal nerve were studied. The first region, (“target”) was chosen to be over the area where the radiosurgical target was located, as determined by the location of the radiosurgical target on the planning software. A second ROI, (“proximal”) was defined as an area proximal to the nerve root entry zone separated with the “target” ROI by at least two voxels. The proximal target still remained outside the nerve entry zone. The sizes of the “target” and “proximal” ROI used in all sets of pre and post-treatment images were equivalent. FA volume was generated from the DTI volume. AD and RD volumes were calculated by custom module developed in-house. The diffusivity statistics were exported from these volumes. “Unaffected” ROI was defined as an ROI in the contralateral, non-symptomatic nerve, with a position and size comparable to the “target” and “proximal” ROIs.

## Results

### Response to treatment (Table 1)

All subjects had significant improvement (S1, S3) or resolution of their pain (S2, S4, S5) at assessment 6 months post-treatment. Two subjects, S1 and S2 were assessed also at one year post-treatment. At that time, S1 had experienced full recurrence of her pain to its baseline. S2 remained pain free.

### Image resolution and ROI definition

**Figure 1:**
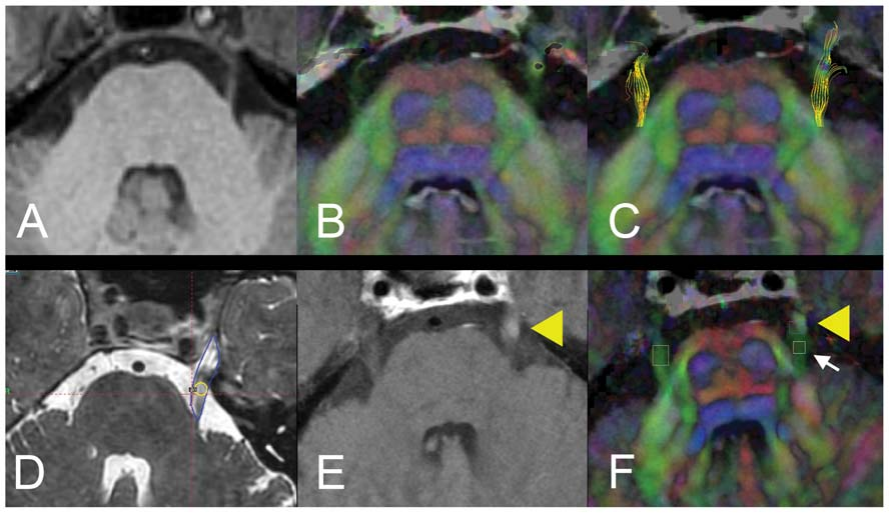
Baseline MR imaging, tractography of the trigeminal nerve, target and ROI definition. Image processing commenced with baseline anatomical 3TMR images (A, axial section, midpontine level). Diffusion tensor images with overlaid colour-by-orientation fibers are shown in B. Reconstructed tracts of the trigeminal nerve onto colour-by-orientation images are shown in C. Panel D depicts the contour of the trigeminal nerve (blue) and location of the radiosurgical shot. Yellow circle denotes the 80% isodose line, representing the “target” of Gamma radiation to the nerve. Panel E shows focal area of post-gadolinium enhancement on the trigeminal nerve (yellow arrowhead), defining the “target” ROI. Panel F shows the location of the “proximal” ROI, proximal to the area of gadolinium enhancement (B, white arrow), and “unaffected” ROI, contralateral nerve.

Figure 1 shows an axial image of the brainstem at the level of the trigeminal nerve. The overlaid tracts of the trigeminal nerve (panel C) demonstrate adequate concordance with the location and size of the tracts. The location of the radiosurgical target, and corresponding selection of ROIs are shown in panels D-F. The small ROI size was not a limitation in trigeminal tract reconstruction or calculation of scalar DTI parameters.

### Changes in DTI parameters after GKRS treatment of the trigeminal nerve

**Figure 2:**
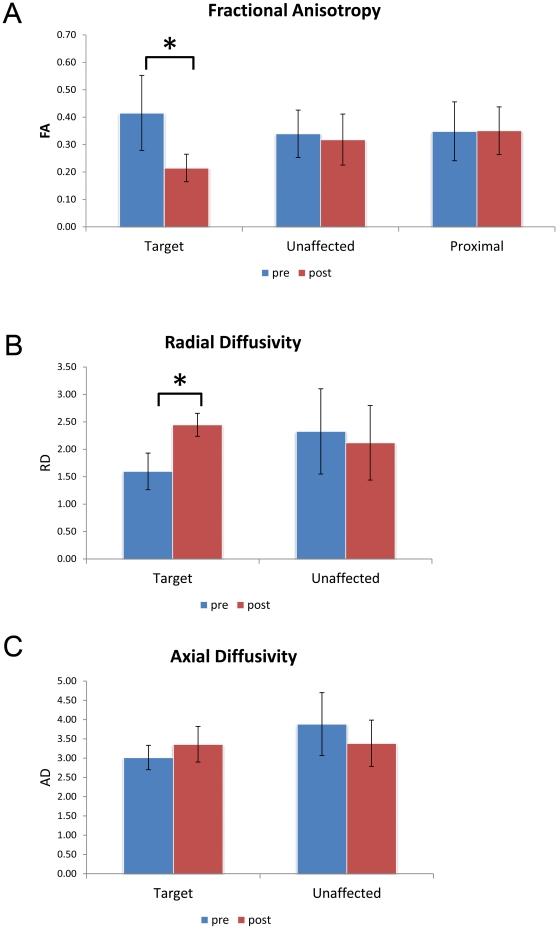
Target ROI is characterized by focal diffusivity changes. Comparison of diffusivities change across all ROIs reveal statistically significant decrease in FA and rise in RD in “target” post-radiosurgery treatment. Rise in RD and non-significant changes in AD point to changes in myelination as main contributor of diffusivity changes. (*denotes statistically significant changes, FA p = 0.027, RD p = 0.002; two-tailed t-test. NS = no statistical significance. RD,AD scalar values are multiplied ×1000 for ease of representation).

To determine whether tractography can detect a focal change in trigeminal nerve anisotropy, the scalar values of FA, RD and AD were studied over different time-points for the selected ROI. “Target” ROI showed a significant decrease in FA after treatment, dropping by 47%, down to 53% of its baseline value (p = 0.027 two-tailed t-test) (Figure 2A, Table 2). “Proximal” ROI showed no significant change in FA, demonstrating that this technique can detect highly focal changes between very small ROI only two voxels apart. The area of focal decrease in FA corresponds to site of delivery of Gamma radiation. Significant rise in RD (increased 55.8% over baseline, paired t-test value = 0.002) but not AD (Figure 2B,C, Table 2), denotes that the proportional contribution of FA is primarily from RD. No changes in the contralateral nerve diffusivities were observed.

**Table 2:** Effect of GKRS treatment on trigeminal nerve diffusivities.

Subjects	Fractional anisotropy			Radial diffusivity		Axial diffusivity	
	Target	Unaffected	Proximal	Target	Unaffected	Target	Unaffected
**S1**	44.6 (83.3)	72.85 (84.4)	101.7	221.7	101.6	111.7	87.7
**S2**	**52.0 (56.61)**	**99.23 (107.4)**	**120.0**	**158.0**	**91.3**	**117.3**	**85.2**
**S3**	**52.9**	**97.14**	**93.3**	**144.7**	**90.0**	**107.1**	**88.9**
**S4**	60.2	101.29	93.9	123.1	82.3	107.8	84.1
**S5**	55.0	98.43	102.5	131.8	92.4	106.8	92.9
**Mean+SD**	53.0±5.6	93.8±11.8	102.3±10.8	155.8±39.1	91.5±6.9	110.2±4.5	87.7±3.4
**p-value**	*0.027	**NS**	**NS**	*0.002	**NS**	**NS**	**NS**

Scalar diffusivity values for each ROI were compared pre and post-treatment, as change from baseline value of 100%. Timepoints for comparison are pre-treatment and 6–7 months post-treatment. S1, S2 timepoints (parentheses) represent values measured at 12 and 14 months respectively. S1 diffusivity values trended towards baseline at 14 months, and paralleled the clinical return to baseline pain levels. S2 remained with stable diffusivity values post-treatment, and no clinical return of trigeminal neuralgia pain. The values of S2 and S3 are bolded, and denote lack of post-treatment MR gadolinium-enhancement. “Target” ROI shows statistically significant decrease in FA and elevated RD (two-tailed t-test. SD = standard deviation, NS = no statistical significance).

### Trigeminal nerve diffusivity is dynamic, but 2D MR changes are not

**Figure 3:**
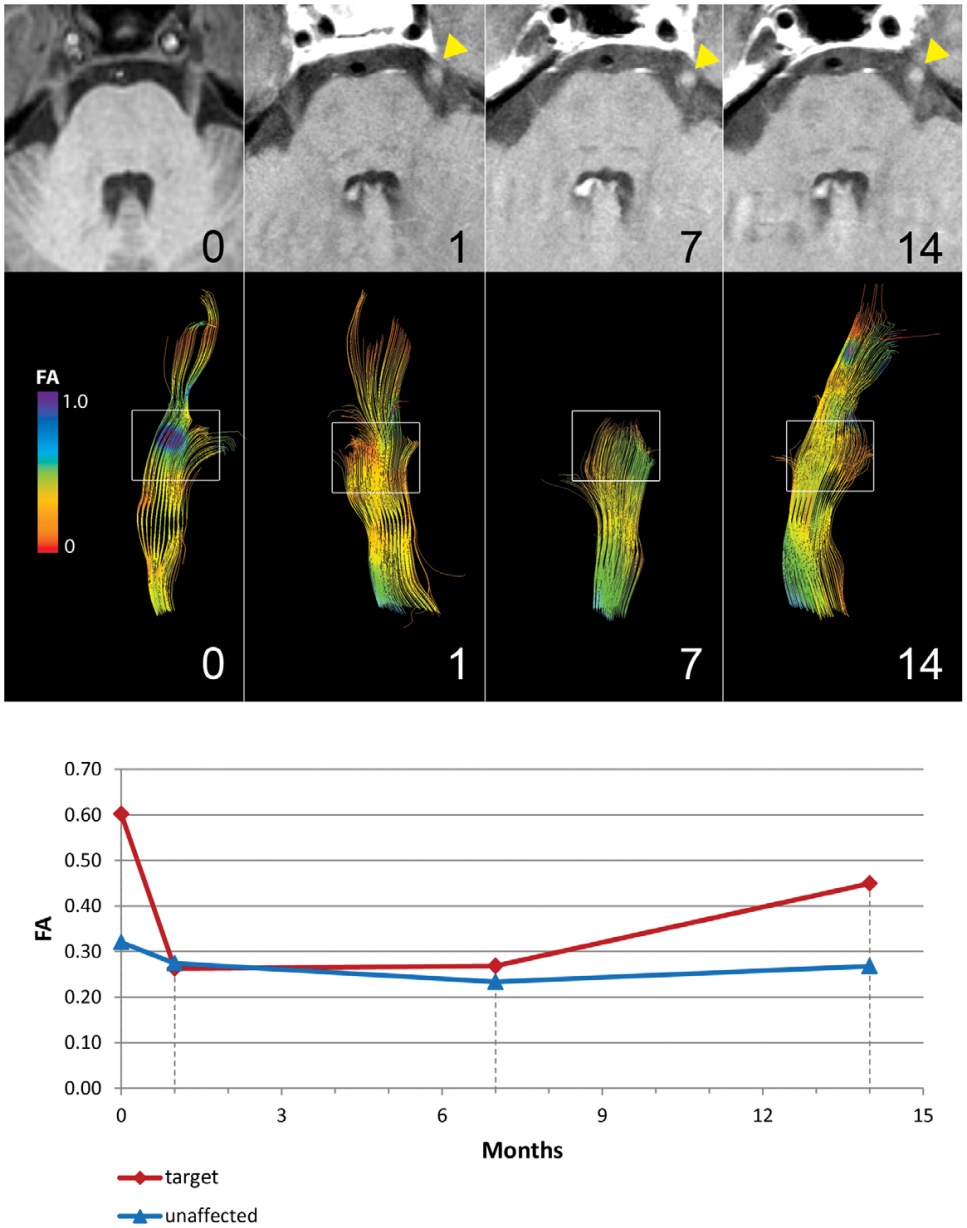
Changes in FA after GKRS treatment are dynamic. Sequential images for subject S1 are shown at 0, 1, 7 and 14 months after Gamma Knife radiosurgery (GKRS) treatment. Top panel depicts serial MR images, showing similar gadolinium enhancement in the midcisternal portion of the nerve after treatment with time (yellow triangles). Middle panel shows reconstructed trigeminal nerve tracts for the same time points. At one month, marked FA decrease is seen in the target area (high FA pre-treatment, blue now appearing as low FA, orange) and tract-pruning due to fall-off of FA value. At 14 months the FA values trends towards baseline, with a longer reconstructed trigeminal segment. The area of low FA has also resolved. Lower panel shows a graph of the scalar values of FA with time. At 14 months, subject S1 has experienced full recurrence of her pain.

To examine possible dynamic changes in the tracts, the pattern of change within the “target” ROI was examined at different time points in subject S1 by reconstructing the tracts at 0, 1, 7 and 14 months post treatment, (Figure 3). An initial sharp decrease in FA was seen at one month, which continued at 7 months. At 14 month this pattern changed, with the most distal fibers now being easily visualized. At this point, the patient's pain had also recurred to baseline. In comparison, gadolinium-enhanced MRs during all time points showed uniform enhancement of the nerve (Figure 3, top panel), demonstrating that tractography is more sensitive than 2D MR imaging and gadolinium-enhancement in detecting changes in the nerve after treatment.

**Figure 4:**
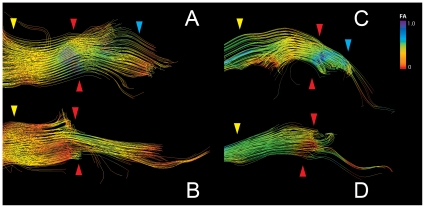
Tractography outlines detailed FA changes in the trigeminal nerve after GKRS treatment. Panels A–D depict the trigeminal nerve tracts pre and post-treatment for subjects S1(A,B) and S2 (C,D). The area between the yellow and blue arrows delineates the cisternal segment, with the yellow arrow being proximal to the brainstem and the blue arrow distal. The red arrow denotes the target area, which corresponds to the region where the greatest change in FA was observed. In S1, FA change affects primarily the outlying fibers of the nerve, while for S2, FA changes are seen in the inferior portion of the cisternal segment of the trigeminal nerve.

To determine whether we could pinpoint the neuroanatomical sub-region where the change in diffusivity occurred, the “target” ROIs was examined in detail. Figure 4A,B depicts two reconstructed trigeminal tracts pre- and post-treatment in subject S1. We observe a near circumferential pattern of change, with apparent preservation of FA in the deep fibers. In subject S2 (Fig. 4 C,D), we observe tract pruning and marked change in FA inferiorly in the nerve. 2D MR assessment alone does not show any notable structural changes.

### Tractography detects changes in the trigeminal nerve in absence of post-treatment MR gadolinium-enhancement

**Figure 5:**
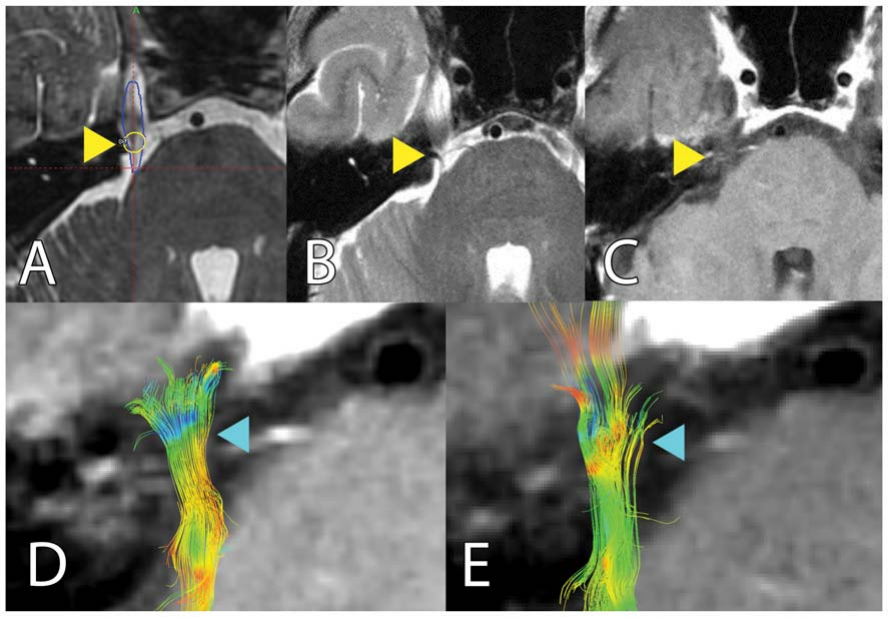
Tractography can detect changes in the trigeminal nerve in the absence of post-treatment gadolinium enhancement: Panels A to E delineate FA changes seen after treatment. Subject S2 did not show post-treatment MR gadolinium enhancement. Panel A shows location of radiosurgical target during treatment planning. Panels B, C depict post-treatment MR and lack of gadolinium-enhancement (yellow arrowhead). Reconstructed trigeminal tracts are shown in panel D (pre-treatment) and E (post-treatment), with clear FA changes in the target area (blue arrowhead).

To investigate whether our technique can detect changes in the absence of gadolinium-enhancement, we compared diffusivity values in the “target” ROI between the subset of subjects that showed gadolinium-enhancement post treatment and those who did not (S2, S3). FA decrease is comparable with the whole group (Table 2), and changes in the “target” ROI could be clearly visualized (Figure 5), pointing to higher robustness and sensitivity of this technique.

### Return of FA to baseline may correlate with pain recurrence

Subjects (S1, S2) had different long-term pain outcomes. To assess whether diffusivity changes correlate with pain outcome, we compared diffusivity values and pain relief with time. In S1, FA dropped after treatment with a subsequent rise to 83% of the baseline 14 months (Table 2). In comparison, S2 (pain free) FA values decreased over 50% after treatment and remained low at 12 months.

## Discussion

In this article we report a novel method of *in vivo* analysis of microstructural changes in the trigeminal nerve to study the effects of focal radiosurgery for TN. We demonstrate for the first time that DTI can detect changes in trigeminal nerve microstructure that correlate with GKRS treatment and effect. These changes consist in 1) highly focal diffusivity changes in the radiosurgical target, 2) increase in RD suggesting a primary effect on myelin, 3) highly sensitive technique that can detect changes in absence of post-treatment MR nerve enhancement and 4)correlation between diffusivity changes and pain, such that recurrence of pain is associated with reversal of FA towards baseline values. The described technique offers a novel improvement in the way in which we study TN and the effect of treatment, since traditionally investigation of this entity have relied mainly on clinical reports, particularly in TN GKRS.

Although changes in FA, AD and RD have been demonstrated in relation to a number of pathological process in the central nervous system (CNS) [17], [18], [21] the relationship between specific diffusivities and clinical outcomes have not been studied in cranial nerve disorders previously. To be able to measure specific diffusivities in small fibers with accuracy clearly opens the door for further applications of this technique.

The applicability of tractography to the study of cranial nerves is novel and few studies have been reported in this area [22], [23]. We recently demonstrated that tractography can be used to delineate the course of the cranial nerves, and that significant information with respect to the DTI parameters can be obtained [19], [24]. Our technical advances allowed us to study the feasibility of detecting changes in the trigeminal nerve after focal radiosurgery for TN.

### Role of tractography in detecting changes in the trigeminal nerve after GKRS

We hypothesized that DTI can detect the changes that occur in the trigeminal nerve after GKRS treatment. To demonstrate a direct relationship, the expected FA changes should reflect the highly focal nature of GKRS and the target location, as calculated during radiosurgical planning and seen in gadolinium-enhanced post-treatment MR (Figure 1D–F). We demonstrate distinct changes in FA in “target” ROI while “proximal” ROI did not change significantly (Figure 2), thereby confirming our hypothesis.

### Proportional changes in RD vs. AD may provide information on the mechanism of action of GKRS

The preferential RD rise suggests that focal radiosurgery affects primarily myelin. This is consistent with our clinical observation of low proportion of permanent sensory changes after GKRS, and lack of trigeminal deficits in this group. Recovery of myelination changes points also towards recurrence of pain. Previous studies have been published on dysmyelination in the CNS and how this process affects DTI parameters [25], [26], however similar findings in the cranial nerves has not been reported. To our knowledge, this is the first report of an *in vivo* model through which the effects of radiation on the trigeminal nerve can be assessed.

### Tractography is superior to gadolinium-enhanced MR in detecting changes in the trigeminal nerve after treatment

The only current imaging modality that can detect the effect of GKRS on the trigeminal nerve consists of post-treatment gadolinium-enhanced MR images, despite there being no direct correlation between enhancement and effect of treatment [14]. We hypothesized that our present technique was able to detect changes in the nerve in the absence of post-treatment enhancement. The magnitude of FA and RD changes for subjects S2, S3 is similar to that of the group (Table 2), indicating that assessment of DTI parameters is a more sensitive and robust test compared with postoperative gadolinium-enhancement alone.

While tractography revealed clear changes in DTI parameters in the same subject across different time-points, we observed no difference in the pattern of gadolinium-enhanced MR (Figure 3). This is significant since it identifies an *in vivo* imaging technique that detects dynamic changes and may correlate with treatment outcome. Additionally, tractography depicts the actual target with greater detail. Whereas gadolinium enhancement always appears circumferential, tractography shows that not all the nerve is equally affected (Figure 4). This has important implications in radiosurgical planning and detection of accuracy of targeting. The dynamic nature of FA changes is also seen in the subset of patients who had post-treatment imaging beyond 12 months. In these, the fall and rise of FA parallels the improvement and subsequent recurrent of pain status, pointing to a possible prognostic value of diffusivity changes (Figure 3, Table 2).

Further study in this area will benefit from imaging, clinical assessment and treatment outcome at multiple time points and larger population. Although ideally histological data would provide additional proof of the effects of focal radiosurgery on the cranial nerve, this is not feasible in the present clinical context. Robust imaging methods of the trigeminal nerve provide the possibility of using tractography for the study of cranial nerve disorders using animal model correlates.

Our study serves as a strong proof of principle of the use of trigeminal tractography and diffusivity assessment in the study of effects of TN treatment. The novel technique provides not only detailed structural views but provides information on neural microstructure. Our findings open the door to new avenues of the study of TN and our approach to the study of pain and other disorders of the cranial nerves.
